# Long-term pulse wave velocity outcomes with aerobic and resistance training in kidney transplant recipients – A pilot randomised controlled trial

**DOI:** 10.1371/journal.pone.0171063

**Published:** 2017-02-03

**Authors:** Ellen M. O’Connor, Pelagia Koufaki, Thomas H. Mercer, Herolin Lindup, Eilish Nugent, David Goldsmith, Iain C. Macdougall, Sharlene A. Greenwood

**Affiliations:** 1 Physiotherapy Department, King’s College Hospital, London, United Kingdom; 2 Department of Renal Medicine, King’s College Hospital, London, United Kingdom; 3 Renal Sciences, King’s College London, London, United Kingdom; 4 School of Health Sciences, Queen Margaret University, Edinburgh, United Kingdom; 5 Department of Renal Medicine, Guys and St Thomas’ Hospital, London, United Kingdom; Weill Cornell Medical College in Qatar, QATAR

## Abstract

**Background:**

This pilot study examined long-term pulse wave velocity (PWV) and peak oxygen uptake (VO_2_peak) outcomes following a 12-week moderate-intensity aerobic or resistance training programme in kidney transplant recipients.

**Method:**

Single-blind, bi-centre randomised controlled parallel trial. 42 out of 60 participants completed a 9-month follow-up assessment (Aerobic training = 12, Resistance training = 10 and usual care = 20). Participants completed 12 weeks of twice-weekly supervised aerobic or resistance training. Following the 12-week exercise intervention, participants were transitioned to self-managed community exercise activity using motivational interviewing techniques. Usual care participants received usual encouragement for physical activity during routine clinical appointments in the transplant clinic. PWV, VO_2_peak, blood pressure and body weight were assessed at 12 weeks and 12 months, and compared to baseline.

**Results:**

ANCOVA analysis, covarying for baseline values, age, and length of time on dialysis pre-transplantation, revealed a significant mean between-group difference in PWV of -1.30 m/sec (95%CI -2.44 to -0.17, p = 0.03) between resistance training and usual care groups. When comparing the aerobic training and usual care groups at 9-month follow-up, there was a mean difference of -1.05 m/sec (95%CI -2.11 to 0.017, p = 0.05). A significant mean between-group difference in relative VO_2_peak values of 2.2 ml/kg/min (95% CI 0.37 to 4.03, p = 0.02) when comparing aerobic training with usual care was revealed. There was no significant between group differences in body weight or blood pressure. There were no significant adverse effects associated with the interventions.

**Conclusions:**

Significant between-group differences in 9-month follow-up PWV existed when comparing resistance exercise intervention with usual care. A long-term between-group difference in VO_2_peak was only evident when comparing aerobic intervention with usual care. This pilot study, with a small sample size, did not aim to elucidate mechanistic mediators related to the exercise interventions. It is however suggested that a motivational interviewing approach, combined with appropriate transition to community training programmes, could maintain the improvements gained from the 12-week exercise interventions and further research in this area is therefore warranted.

**Trial registration:**

study number: ISRCTN43892586.

## Background

Kidney transplantation improves survival, quality of life, and is more cost-effective than other treatment for end stage renal disease (ESRD) [1, 2]. Despite this, the incidence of cardiovascular disease (CVD) remains 3 to 5 times higher in kidney transplant (KTx) recipients than the general population [3]. This is thought to be due to a high prevalence of comorbidities, physiological stressors experienced whilst receiving haemodialysis [1], episodes of rejection, or use of immunosuppressant medications [3, 4]. Therefore, cardiovascular risk (CVR), and potential interventions that reduce it, is of upmost importance in this patient population.

Low physical activity levels are associated with metabolic syndrome, CVD, and abnormal glucose tolerance in KTx recipients [5, 6]. In contrast, higher levels of physical activity in KTx recipients have been associated with a reduction in incidence of CVD and all-cause mortality [6, 7]. Several small studies have suggested short-term exercise related benefits in kidney transplant patients [8–11]. However engagement in long-term behavioural change appears challenging. A recent systematic review [12] demonstrated medium level evidence of motivational interviewing techniques to increase physical activity in patients with chronic disease. Approaches to facilitate engagement and promote lifestyle behavior change may provide long-term engagement with physical activity, and result in reduction in CVD risk factors.

Pulse wave velocity (PWV), the gold standard measure of arterial stiffness [13], has been shown to predict mortality in patients with essential hypertension [14], end-stage kidney disease [15] and more recently it has been shown to be a strong predictor of CVD in KTx recipients [16]. Studies in haemodialysis (HD) patients have shown that three months of Aerobic Training (AT) [17, 18] or resistance training (RT) [19] can induce improvements in PWV. A recently published Randomised Controlled Trial (RCT) from our research team [11], reported significant improvements in PWV immediately post 12 weeks of either supervised aerobic or resistance training, when compared with usual care (UC), in KTx recipients. Long-term outcomes are of utmost importance when evaluating the clinical importance of interventions. Small exercise studies by Mustata et al [17] and Toussaint et al [18], in HD patients, have shown that following one-month cessation of an exercise intervention, PWV values revert back to pre-intervention baseline levels.

Reduced cardiorespiratory fitness, as measured by peak oxygen uptake (VO_2_peak) has been shown to predict survival in haemodialysis patients [20] and KTx recipients [21]. Four RCTs have shown improvements in VO_2_peak immediately post exercise intervention in KTx recipients [8–11]. No studies to date have investigated the long-term effects of RT or AT interventions on PWV or VO_2_peak in KTx recipients after intervention has ceased. A study by Painter et al [8] did report gains in VO_2_peak at 12 months. However, this was with a 12-month aerobic home exercise programme. The purpose of this pilot study was to investigate the long-term effects of a 12 week supervised aerobic or resistance training intervention at 9-month follow-up.

## Methods

### Ethics statement

Ethical approval, in accordance with the Helsinki Declaration, was sought and obtained from the London and St Giles Ethics committee, and Research & Development teams at both sites granted approval for the ExeRT study on the 20^th^ of December 2012 (REC number: 12/LO/1644, IRAS project ID: 75391). An amendment to include VO_2_peak assessment at 9-month follow-up was approved on the 11^th^ of June 2014 date by the London and St Giles Ethics Committee, and Research and Development teams at both sites. The study was registered and confirmed for inclusion on the UKCRN portfolio on the 20/11/2012. The link to the ISRCTN registration was not received from the UKCRN until the 30/07/2014, and the trial was therefore registered on the 31/07/2014. As this was an unintentional process error, an editorial note is in situ on the trial website (www.isrctn.com; study number: ISRCTN 43892586). The study was not amended in any way after initial registration with the UKCRN. The authors confirm that all ongoing and related trials for this intervention are registered.

### Participants

This current study reports the 9-month follow-up results of the Exercise in Renal Transplant (ExeRT) study cohort [11]. This study was a single-blind parallel, randomised controlled trial. 60 participants were recruited and consented to the original ExeRT pilot study [11] from March 2013 to October 2014. Potential participants were given verbal and written information, and consented two weeks later if willing to participate.

Participants were included if they were 18 years of age or older, able to provide written consent, and if they had received a kidney transplant in the preceding 12 months. They were excluded if they were unable to walk 50 metres independently, were pregnant, had participated in a structured exercise programme in the past six months, or if they had any medical condition that would preclude participation.

A sample size of 37 in each group will have 80% power to detect a mean difference of 1.8 m/sec [17] in PWV between either exercise group (AT and RT) or the UC group.

Assuming that the common standard deviation is 2.7, (effect size = 0.7), using a one-way analysis of variance with a 0.05 two-sided significance level. Adjusting this for an anticipated attrition rate of 20% gives a final sample size of 132 (44 in each group). The published Minimal clinically important difference (MCID) for PWV is a reduction of 1 m/s [16]. The ExeRT pilot study recruited 60 participants.

Participants were randomised after baseline assessment to either 12 weeks of supervised aerobic training, resistance training or usual care by computer randomization. This was performed by an independent member of the research team. Participants were asked not to disclose their allocation during assessments, ensuring blinding of research assistants. All time point assessments baseline, 12 weeks and 9-month follow-up post intervention (12 month time-point) were conducted between April 2013 and July 2015. All 9-month follow-up assessments were collected within a 14-day window and in a single research visit at a clinical research facility. The measurements were collected at the same time of day, and in the same order, as previous baseline and 12-week assessments.

### Primary outcome

The primary outcome for this study was PWV. PWV was measured using the Vicorder system (Skidmore Industries, UK) at 9-month follow-up and compared to measures recorded at baseline and 12 weeks. Arterial stiffness was assessed at the systemic region (carotid-femoral PWV), the gold standard method [13]. Conditions for assessment, as stated by the expert consensus statement by Laurent et al [22], were adhered to for all measurements. The measurement protocol by Hickson et al [23] was used mathematically removing the additional femoral segment from the Vicorder standard protocol, to correct for any inherent bias at high arterial PWV, and also accounting for participants mean blood pressure value. The average of 3 measurements (of 20 consecutive signals) was recorded at each time point.

### Secondary outcomes

#### Cardiorespiratory fitness

VO_2_peak was determined during an incremental recumbent cycling exercise tolerance protocol. Breath-by-breath gas exchange was measured using a Cortex metalyser system, (MetaLyzer^®^ 3B cardiopulmonary exercise testing equipment, Cortex, Germany) calibrated prior to each patient assessment. The exercise protocol started with 3 minutes of cycling without resistance, and this increased by 15 watts/minute until one of the following occurred: i) a plateau in oxygen uptake ii) attainment of a respiratory exchange ratio of 1.15 or greater, or iii) patient request to stop. The average oxygen uptake of the final 20 seconds of the test was recorded as the VO_2_peak. ECG monitoring, blood pressure, and heart rate were continuously recorded throughout the incremental test to ensure participants safety.

#### Anthropometric measures and resting blood pressure

Body weight assessment was recorded, and height was measured using a calibrated stadiometer. After sitting quietly for 5 mins, resting blood pressure was recorded in triplicate, with a 1-min interval between measurements, using an automated sphygmomanometer (Tango; SunTech Medical, Oxfordshire, UK). The average of the 3 readings was recorded.

### Exercise intervention

Participants randomized to either intervention group (AT or RT) were offered twice-weekly supervised, and once-weekly home-based, individually tailored exercise training for 12 weeks at King’s College Hospital (KCH) or Guys and St Thomas’ Hospital (GSTT). Both interventions (AT and RT) were provided at hospital outpatient gyms by a senior renal physiotherapist and a physiotherapy assistant. Once a week, 30-minute, physiotherapist led patient education was also provided. Topics included; the role of exercise in renal disease, personalizing exercise plans, goal settling, long-term community exercise options, problem solving and overcoming barriers and a question and answer session. Motivational interviewing techniques [24] were utilized throughout the supervised exercise interventions, and to encourage engagement with the self-management exercise activity plans. At completion of 12-week interventions, all AT or RT participants were encouraged to engage with self-managed community exercise pathways and remain physically active. After this facilitation to community exercise at 12 weeks, patients received no further intervention. Participants in the usual care group were not provided with any specific exercise guidance, they received general exercise encouragement from nursing staff or nephrologists at routine clinic appointments. For further information on the supervised intervention see Greenwood et al [11].

### Statistical analysis

This current study reports the long-term follow-up data of the 42 remaining ExeRT study participants who were assessed at 9-month follow-up. The results were compared with baseline and 12-week data from the ExeRT study [11]. Descriptive data were recorded including baseline characteristics for dropouts. Statistical analysis of outcomes was completed using an as-treat analysis [25] and SPSS version 22 (PASW Chicago 11). A significance level of p<0.05 was accepted. Before statistical testing, data was checked for normality. ANCOVA analyses covarying for time on dialysis pre-transplant, age and baseline values were employed to detect mean between-group differences at 9-month follow-up. Paired t-tests with Bonferroni correction (set at 99% error) were utilised to assess within-group differences in data from 12 weeks to 9-month follow-up assessment.

## Results

**Fig 1:**
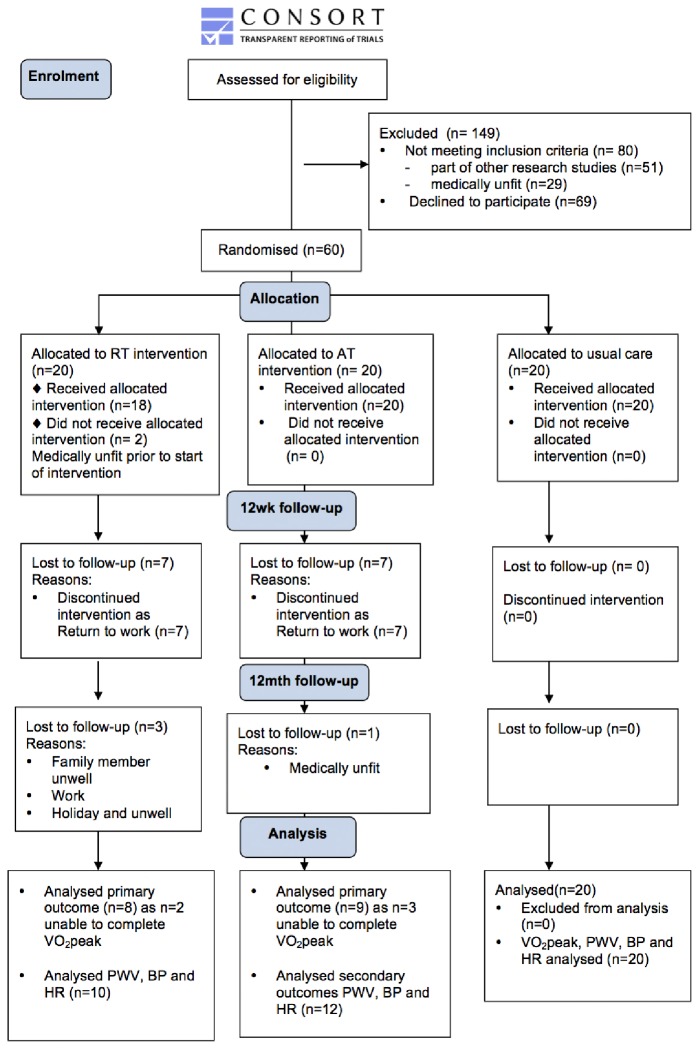
CONSORT flow diagram. Demonstrating flow of participants throughout the study at baseline, 12 weeks, 12 months (9-month follow-up), and those included in final analysis.

42 participants remained in the study at 9-month follow-up assessment (Fig 1), with a 30% attrition rate. There were 27 males and average age was 51.8±12.5 years, with a mean transplant vintage of 28.6±19.6 weeks. Time on dialysis prior to transplant was 30 [5.75–54.25] months. PWV was assessed at 9-month follow-up in all 42 patients. VO_2_peak was assessed in 37 of the 42 subjects at 9-month follow-up assessment. 5 subjects were unable to complete VO_2_peak testing at 9-month follow-up due to musculoskeletal knee pain (AT = 1, RT = 2), and 2 participants (AT) were awaiting cardiac investigations for conditions unrelated to the intervention. The modified consort diagram in Fig 1 shows the participant flow in the study. Participant characteristics including dropouts from 12 weeks to 9-month follow-up (n = 46) are reported in Table 1.

**Table 1:** Participant baseline demographics and 9-month follow-up outcomes.

Variable	Total sample (n = 46)	UC (n = 20)	AT (n = 13)	RT (n = 13)
**Age (Mean years)**	51.8±12.5	49.5±10.6	53.9±10.7	54.6±10.6
**Males**	27 (58.7)	10 (50)	10 (77)	7 (54)
**Ethnicity**	W = 24 (52) B = 19 (41) A = 3 (6)	W = 10 (50) B = 9(45) A = 1(5)	W = 7 (54) B = 5(38) A = 1(8)	W = 7(58) B = 5(38) A = 1(8)
**Transplant vintage (Mean weeks)**	28.6±19.6	27.7±17.4	26.5±21.1	32.1±22.3
**Transplant characteristics**	1^st^ = 35(76.1) 2^nd^ = 5(10.9) 3^rd^ = 2 (4.3) SPK = 4 (8.7)	1^st^ = 16 (80) 2^nd^ = 2 (10) 3^rd^ = 1 (5) SPK = 1 (5)	1^st^ = 11(84.6) 2^nd^ = 2 (5.4)	1^st^ = 8 (61.5) 2^nd^ = 1 (7.7) 3^rd^ = 1 (7.7) SPK = 3(23.1)
**Transplant type**	L = 18 (39.1) C = 28 (60.9)	L = 6 (30) C = 14 (70)	L = 8 (61.5) C = 5 (38.5)	L = 4 (30.8) C = 9 (69.2)
**Time on dialysis (Median [IQR])**	30 [5.75–54.25]	41.5 [18.5–52]	34.0 [9.8–58.3]	11.5 [0–56.7]
**Steroid therapy**	46 (100)	20 (100)	13 (100)	13 (100)
**Calcineurin Inhibitor immunosuppression therapy**	46 (100)	20 (100)	13 (100)	13 (100)
**Statin therapy**	46 (100)	20 (100)	13 (100)	13 (100)
**HTN**	20 (43.5)	7 (35)	9 (69.2)	4 (30.8)
**Diabetes**	T1 = 8 (17.8) T2 = 9 (26)	T1 = 5 (25) T2 = 1 (5)	T2 = 2 (15.4)	T1 = 3 (25) T2 = 6 (50)
**Hospitalisations**	14 (30.4)	8 (40) Planned = 1 Unplanned = 7	4 (15.4) Planned = 1 Unplanned = 3	2 (15.4) Planned = 1 Unplanned = 1
**Rejection episodes**	6 (13%)	3 (15%)	1 (7.7%)	2 (18.2%)
**NODAT**	6 (13%)	2 (10%)	4 (30.8)	4 (30.8)
**CVE**	2 (4.3%)	0	1 = DO	1 = DO
**Deaths**	0	0	0	0

Means and Standard deviations are presented for continuous data. Frequency numbers and proportional percentages are shown for categorical variables and events. Length of time on dialysis prior transplant is expressed in Median with inter quartile ranges. KTx = kidney transplant, Tx = transplant, HTN = hypertension, NODAT = New onset of Diabetes after transplant, CVE = cardiovascular event, W = white, B = black, A = Asian, 1st = 1st Tx, 2nd = 2nd Tx, 3rd = 3rd Tx, SPK = simultaneous pancreas kidney Tx, L = live donor and C = cadaveric donor.

There were 14 hospitalisations (30%) across the sample, 1 planned and 7 unplanned admissions in the UC group, 1 planned and 3 unplanned in the AT group and 1 planned and 1 unplanned admission in the RT group. There were two cardiovascular events (one in the AT and one in the RT group). One of these participants (RT group) was considered to be non-compliant with all medications, and had a myocardial infarction that was deemed to be unrelated to the exercise intervention. The other participant (AT group) was non-compliant with the exercise intervention and was investigated for a pre-existing cardiac issue. There were six episodes of rejection, six participants developed new onset of diabetes after transplant (NODAT), and there were no deaths across the sample, see Table 1.

### Primary outcome (PWV)

**Fig 2:**
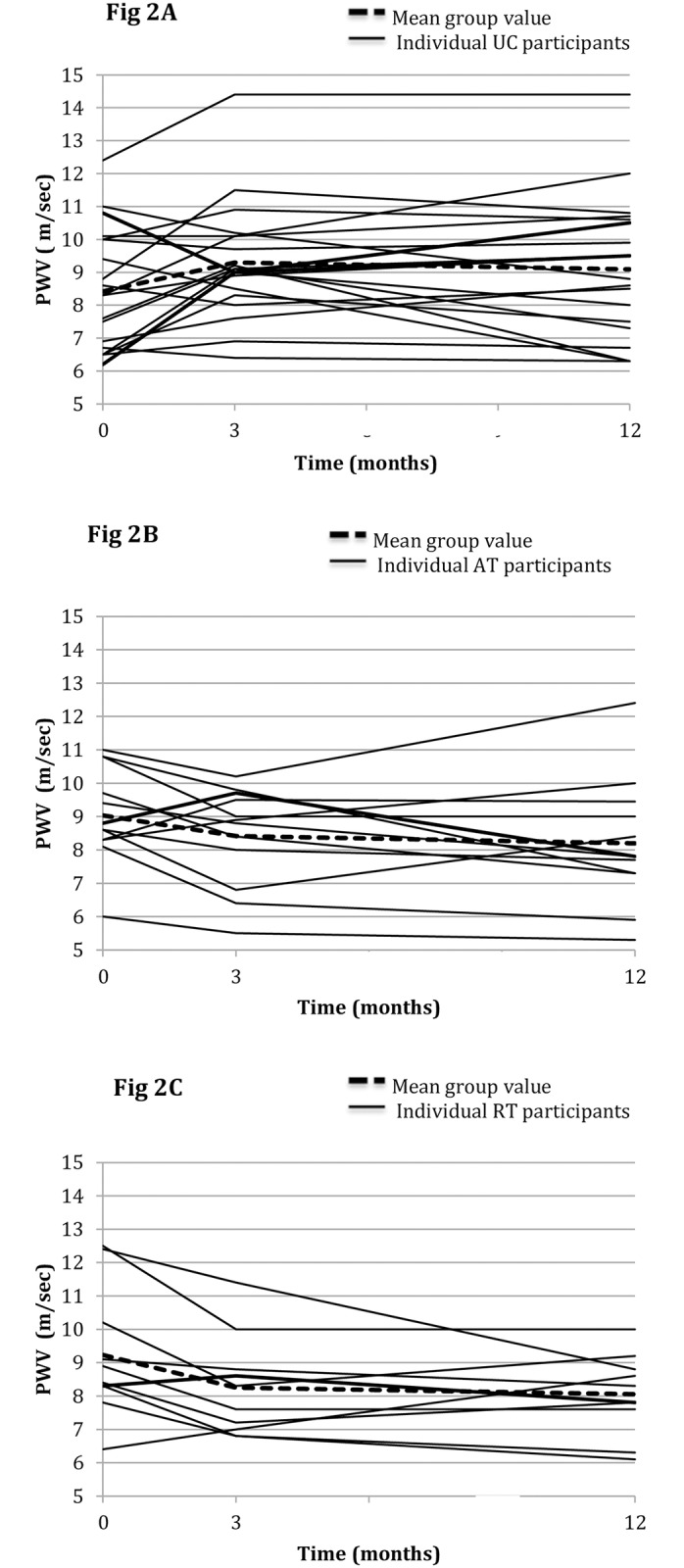
Individual PWV results (m/sec) in each group at baseline, 12 weeks and 12 months (9-month follow-up). **(A)** UC group (n = 20), **(B)** AT group (n = 12), and **(C)** RT group (n = 10).

Table 2 below depicts data at baseline, 12 weeks, 9-month follow-up assessment and ANCOVA analysis. Data at baseline and 12 weeks are reproduced with permission, from Greenwood et al [11]. There were no significant within-group changes in PWV from the 12-week to 9-month follow-up assessment. However, ANCOVA analysis revealed a significant mean between-group difference in PWV of -1.30 m/sec (95%CI -2.44 to -0.17, p = 0.03) between the RT and UC groups. When comparing AT and UC groups at 9-month follow-up, the mean difference was approaching significance, -1.05m/sec (95%CI -2.11 to 0.017, p = 0.05). Fig 2 depicts individual PWV results across the three time points in all participants remaining in the study at 9-month follow-up assessment (n = 42).

**Table 2:** Long-term outcomes at 9-month follow-up-, and results of 9-month follow-up ANCOVA analysis.

Variable	Baseline (n = 46)			12weeks (n = 46)			12months (n = 42)			9-month follow-up assessment ANCOVA			
	UC(n = 20)	AT (n = 13)	RT (n = 13)	UC (n = 20)	AT (n = 13)	RT (n = 20)	UC (n = 20)	AT (n = 12)	RT (n = 10)	Groups	Between -group X diff	95% CI	p-value
**Arterial stiffness**													
PWV (m/sec)	8.9±2.3	9.0±1.4	9.1±1.8	9.4±2.3^a^	8.4±1.6^a^	7.7±1.4^a^	8.9±1.6	8.2±1.9	8.1±1.2	**AT/UC**	-1.05	-2.11, 0.017	0.05*
										**RT/UC**	-1.30	-2.44, -0.17	0.03*
**Cardio pulmonary exercise test secondary variables**													
Relative VO_2_peak (ml/kg/min)	11.8±3.0	12.3±4.8	14.1±4.0	12.8±3.0	15.1±5.3^a^	16.8±3.9^a^	15.9±5.2	16.2±6.4	13.4±3.9	**AT/UC**	2.20	0.37, 4.02	0.02*
										**RT/UC**	0.89	-1.10, 2.89	0.39
Absolute VO_2_peak (L/min)	0.8±0.2	0.9±0.4	1.1±0.6	1.0±0.4	1.1±0.3^a^	1.2±0.6^a^	1.1±0.5	1.2±0.6	1.1±0.3	**AT/UC**	0.08	-0.09, 0.25	0.36
										**RT/UC**	0.07	-0.12, 0.26	0.47
RER	1.0±0.0	1.1±0.1	1.0±0.1	1.0±0.0	1.1±0.2	1.0±0.1	1.1±0.1	1.0±0.3	1.0±0.1	**AT/UC**	-0.07	-0.22, 0.09	0.41
										**RT/UC**	-0.002	-0.17, 0.17	0.97
Body mass (kg)	71.6±10.8	76.3±15.6	79.4±13.8	76.9±12.1^a^	78.5±16.9	79.5±14.8	72.5±15.9	77.8±14.8	82.3±12.8	**AT/UC**	0.29	-3.51, 4.10	0.87
										**RT/UC**	3.29	-0.73, 7.16	0.09
Resting SBP (mmHg)	133.9±11.6	136.8±14.4	135.9±13.3	135.7±12.4	134.9±12.2	136.0±13.3	132.2±11.7	134.6±15.5	136.9±13.2	**AT/UC**	-0.8	8.78, 7.16	0.83
										**RT/UC**	2.08	-6.45, 10.59	0.62
Resting DBP (mmHg)	71.4±8.4	77.6±13.7	81.7±12.5	73.5±8.7	75.1±14.0	84.7±10.7	76.9±8.4	76.6±10.9	74.2±10.6	**AT/UC**	-1.93	-8.39, 4.52	0.54
										**RT/UC**	-3.79	-10.66, 3.07	0.26

(n = 46 at baseline for PWV, weight, SBP, DBP and all V02peak related variables. N = 42 at 12months for PWV, weight, SBP and DBP. VO2peak related data n = 37 at 12 months).

Values are expressed as means and standard deviations. VO2peak = peak oxygen uptake, CPET = cardiopulmonary exercise test, HR = heart rate, SBP = systolic blood pressure, DBP = Diastolic BP. Significance p<0.05.

^a^ Indicates within-group significance from baseline to 12 weeks. There were no significant within-group differences in variables from 12 weeks to9-month follow-up assessment.

* significant mean between-group difference from ANCOVA analysis (allowing for mean baseline values, age and length of time on dialysis pre-transplantation).

### Secondary outcomes

**Fig 3:**
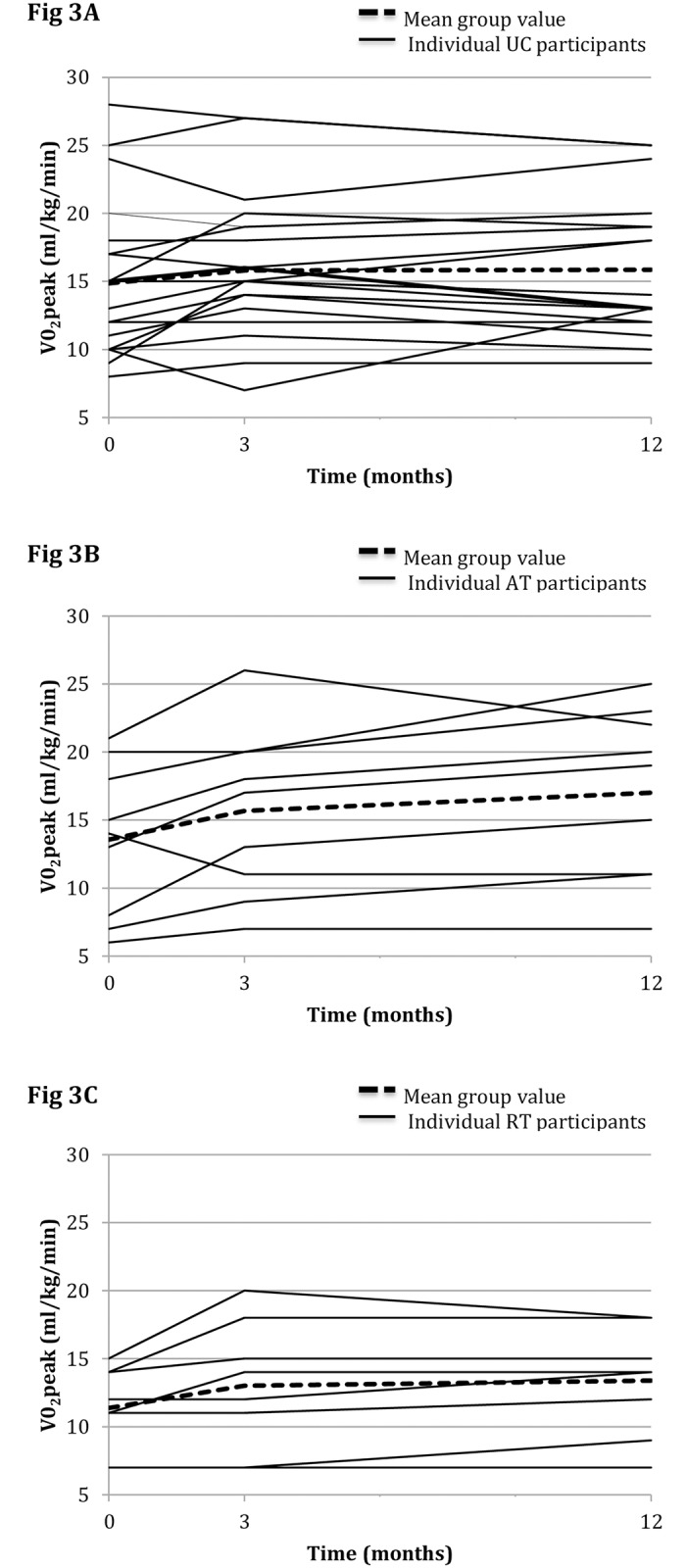
Individual relative VO2peak values (ml/min/kg) in each group at baseline, 12 weeks and 12 months (9-month follow-up). **(A)** UC group (n = 20), **(B)** AT group (n = 9), and **(C)** RT group (n = 8).

There were no significant within-group differences in the AT, RT or UC groups from the 12-week to the 9-month follow-up assessment in absolute or relative VO_2_peak values, Respiratory exchange ratio (RER), resting blood pressure or weight (Table 2). On ANCOVA analysis, there was however a significant mean between-group difference in relative VO_2_peak of 2.2 ml/kg/min (95% CI 0.37 to 4.03, p = 0.02) between the AT and UC groups. The mean between-group difference in relative VO_2_peak of 0.89 ml/kg/min (95%CI -1 to 2.9, p = 0.4) between the RT and UC groups was not statistically significant. (See Table 2 and Fig 3). There were no significant between-group differences in resting blood pressure or weight scores between AT or RT and UC at 9-month follow-up assessment.

## Discussion

This is the first study to examine the potential long-term outcomes in PWV and VO_2_peak outcomes following a supervised 12-week AT or RT intervention in new KTx recipients. 9-month follow-up PWV results suggest a significant between-group difference of -1.30 m/sec when comparing the resistance training group with usual care. A minimal clinically important difference (MCID) of a reduction of 1m/sec in PWV has been strongly associated with decreased cardiovascular disease risk in KTx recipients [16]. It is of note that the between-group difference in PWV of -1.05m/sec, when comparing the AT and UC group, was a MCID result although this did not achieve statistical significance (p = 0.05).

This pilot study offered patients 12 weeks of supervised training, followed by 9 months of self-managed physical activity. In contrast to previous studies in haemodialysis patients, where PWV values revert back to baseline values after only one month cessation of the supervised exercise intervention [17, 18], this current study demonstrates a significant mean difference that exceeds the MCID even at 9-month follow-up. Interventions in this current study were provided by specialist renal physiotherapists who were trained in motivational interviewing [24]. Patient engagement, with transition from supervised training to self-managed physical activity programmes, was facilitated by these trained individuals. The importance of specialist exercise personnel to sustain positive exercise behavior has been highlighted in the literature [26–29]. A weight management programme delivered by specialist renal physiotherapists, which utilises the same self-management exercise counseling techniques, demonstrated improved exercise capacity and functional ability after 12 months of self-managed physical activity [30].

The effects of resistance training on endothelial function and arterial stiffness remain largely unexplored in the kidney transplant literature. The plausible mechanisms responsible for improvements in PWV with this form of exercise intervention, as demonstrated by 12 week and 9-month follow-up results, remain unclear. It has been hypothesised that improvements in PWV following 3 months of exercise intervention in haemodialysis patients [17–19] may be due to reductions in systolic blood pressure following exercise interventions. Whilst this study did not reveal significant differences in blood pressure between interventions and usual care, this may be explained by the tightly controlled anti-hypertensive regimes in post-transplant care. Although muscle strength was not assessed at 9-month follow-up assessment, 12-week analysis [11] did reveal a significant improvement, and association in muscle strength and PWV, when comparing RT to UC. It is recognized that the RT group had a shorter median time exposure to haemodialysis therapy when compared with UC or AT participants. Whilst this has been controlled for in statistical analyses, it is acknowledged that increased exposure to haemodialysis-related uraemic symptoms has a reported negative effect on vascular health [31], and this must be taken into consideration when interpreting the results. Due to a number of dropouts by the 9-month follow-up point, there was a resultant imbalance in gender distribution in the AT group (Table 1). Although there has been several investigations into the effect of gender as a determinant of PWV, it is suggested that this is not an independent determinant for this outcome [32]. Future studies should assess the physiological mechanisms responsible for change in PWV in this patient population, such as endothelial function.

At 9-month follow-up, there was a significant between-group difference in relative VO_2_peak when comparing AT with UC, however, there were no significant between-group differences found in absolute VO_2_peak values at this time point. It is likely that a lack of significant difference in absolute VO_2_peak values when comparing either intervention group with usual care may be due to the non-significant weight increase seen in both intervention groups (Table 2). Additionally, there were more males than females in the AT group and as there is a possible gender bias with regard to VO_2_peak outcomes [33], caution should be taken with interpretation of these results.

The baseline VO_2_peak values in this current study are significantly lower than those previously reported in other randomized controlled trials in kidney transplant recipients [8–10]. The values also fall below the previously reported value of 17.85ml/kg/min [21], which is associated with a survival advantage in kidney transplant recipients. The low baseline values are possibly reflective of a pragmatic recruitment of any kidney transplant patient who attended a UK National Health Service clinic, and who fulfilled the inclusion criteria for the study, rather than targeting those individuals who were already physically active.

To our knowledge, this is the first long-term follow-up study to examine PWV and VO_2_peak in KTx recipients following an un-supervised period of self-managed physical activity. A 12-month, unsupervised, exercise study by Painter et al [8] reported a 42% attrition rate at 12 months. This current pilot study reports an attrition rate of 30% at the 12 month time point (9-month follow-up assessment), which is comparable to the exercise literature in other stages of the CKD trajectory [34]. It is acknowledged however that the dropouts from the current study, which were limited to the intervention groups, may have resulted in a selection bias that could have influenced the results.

Further limitations include; small sample size, and no formal assessment of patient engagement and adherence with the self-managed exercise recommendations. Assessment of muscle mass, body composition and endothelial function could have also provided insight into mechanisms responsible for change in PWV and relative VO_2_peak outcomes, and should be encouraged in further research to elucidate potential mechanisms for change in exercise related outcomes.

## Conclusion

Possible long-term gains in PWV and VO_2_peak outcomes for new kidney transplant recipients with only 12 weeks of supervised exercise training appears to be feasible. However, large-scale investigations into which type of exercise intervention is most effective for improving outcomes in the short, and longer term are required to confirm this. Please refer to S1 and S2 Figs for study protocol and consort checklist. Refer to S1 Table for raw data.

## Supporting information

S1 Fig study protocol. (DOCM)

S1 Table supplementary raw data. (XLSX)

S2 Fig Consort checklist. (DOC)
