# Supplementary material for: Long-term pulse wave velocity outcomes with aerobic and resistance training in kidney transplant recipients – A pilot randomised controlled trial
Source: PLoS One. 2017 Feb 3;12(2):e0171063. doi: 10.1371/journal.pone.0171063 (PMC5291475; doi:10.1371/journal.pone.0171063)
Supplement: S1 Fig — (DOCM) [file pone.0171063.s001.docm]

**Protocol exert study NIHR Fellowship Masters Clinical Research**

**Ellen O’Connor**

**1. Title and lay description and background:**

***The long-term follow-up effects of 12-weeks of resistance training or aerobic training on physical activity levels and cardiorespiratory fitness in kidney transplant recipients.***

Physical activity and exercise are important in many chronic diseases; to not only improve functional capacity, but also obesity and cardio vascular risk (CVR). Patients with Chronic Kidney Disease (CKD) have been shown to have lower levels of physical activity, cardio respiratory fitness and functional capacity(1-3) and high CVR (4). Kidney Transplant (KTx) recipients have been shown to increase both physical activity levels and cardio respiratory fitness following transplantation, yet they do not reach that of the normal, healthy population (5, 6). Research has shown that these factors can be improved with exercise intervention, however this has not yet been incorporated into clinical practice. This may be due to discrepancies between exercise dosage in present studies with varied frequency, duration and modality of exercise (aerobic, resistance or combined) interventions (4, 7). The purpose of this current study is to evaluate the long-term effects of a specific and appropriated dosed Aerobic training (AT), resistance training (RT), versus usual care on arterial stiffness, (measured by pulse wave velocity), and cardio respiratory fitness (measured by VO_2_peak). The project aim is assess feasibility of this approach and provide data for further studies in this field.

**2. Purpose of proposed investigation:**

The benefits of regular exercise and physical activity (PA) are well documented and recommended for many chronic diseases. Patients with chronic kidney disease have been found to have not only deficits in cardio respiratory fitness (8), physical activity (2, 4), but also skeletal muscle wasting (9) and the ability to perform activities of daily living (1, 10). For kidney transplant (KTx) recipients, this process is exacerbated by the systemic effects of immunosuppressant’s, uremic toxicity (7), fatigue (11, 12) and previous deconditioning and low physical activity levels.

KTx recipients improve cardio respiratory fitness and physical activity levels, however they do not reach levels of the healthy population (5, 6, 11, 13). VO_2_ Peak is a measure of cardio respiratory fitness, and has been used as a marker for mortality in the CKD population (8), and is lower in KTx recipients compared with normal population (9, 14). Studies have demonstrated a further increase of VO_2_ Peak with exercise intervention however these levels, do not reach that of normal, healthy controls (6, 15-17). No known Randomised Controlled Trials to date have investigated the long-term effects of exercise on cardiorespiratory fitness (VO_2_peak) or arterial stiffness after a period of exercise intervention cessation. With the majority of exercise studies in the KTx population have varied in exercise intervention dosage, duration and exercise modality (AT, RT or combined AT &RT) (4, 7), with few studies have investigated purely Aerobic Training (AT) or Resistance training (RT) in KTx recipients. The purpose of current study is to evaluate the long-term effects of RT and AT versus usual care on VO_2_ peak and arterial stiffness.

This current study, is a follow-up study of the ExeRT (exercise in renal transplant) trial, an internal pilot randomised control trial for 60 patients with outcomes measured at baseline, 12 weeks and 9-month follow-up (18). This current study will include data from baseline, 12 weeks and 9-month follow-up to examine the long-term effects of aerobic, resistance training or usual care on VO_2_ Peak and arterial stiffiness (pulse wave velocity) in participants (KTx recipients),. The project aims to examine feasibility of this approach, and provide data to inform further studies.

**4. Study participants (inclusion and exclusion criteria; recruitment):**

Recruitment and Sampling

60 Study participants are already recruited and consented to the exert study from Kings College Hospital and Guys and St Thomas’ outpatient kidney transplant clinics. Participants were randomised to either 12weeks of supervised AT, RT or UC, assessor and analyser of data blinded to allocation. This current study includes those patients who have 12 week and 12month data that where available during the data collection period (September 2014 to July 2015).

As VO_2_ peak measured at 9-month follow-up assessment is an additional outcome to the original study, patient will be asked to complete a second consent form.

Inclusion criteria:

- All patients undergoing renal transplant
- Male or female
- Aged >18 years
- Written informed consent

Exclusion criteria:

- Unstable cardiac disease
- Requiring support for ambulation less than 20m
- Vasculitis
- Active proliferative diabetic retinopathy,
- Severe osteodystrophy
- Uncontrolled diabetes
- Psychiatric illness, including anxiety, mood and untreated eating disorders

**5. Plan of investigation, including research methodology proposed:**

Hypotheses:

1. H0= There is no difference in mean PWV when comparing the three groups (AT, RT and UC) across the three time points

H1= There is a significant difference in PWV when comparing the three groups (AT,RT and UC) across the three time points

1. H0= There is no difference in mean VO_2_ peak when comparing the three groups (AT, RT and UC) across the three time points.

H1= There is a significant difference in VO_2_ peak when comparing the three groups (AT, RT and UC) across the three time points.

Study Design:

Prospective internal pilot RCT with two Rx arms (AT and RT) compared with UC.

Power calculation:

The exert study was designed as an internal pilot with 60 study participants. This is a feasibility study; therefore no power calculation was completed.

Objectives:

*Primary end point*

Pulse wave velocity, measured sing the Vicorder system (Skidmore Industries, UK) at carotid and femoral points using Laurent et al (2006) procedure and Hickson et al (2009) calculation of arterial path length.

*Secondary end points*

Cardiorespiratory fitness is measured by VO_2_ peak using the Cortex MetaLyzer 3B metabolic system with a recumbent stationary bicycle.

Study Logistics:

- Full ethics and R and D approval has been granted (02/02/13)
- An amendment to ethics was approved (11/06/14) for the additional VO_2_ peak measurement at 12/12.
- Baseline data will include patients demographics, group allocation, medical history, drug history, cause of chronic kidney disease, date and type of transplant, assessment of V02 peak and PWV
- 12 week data will include assessment of V02 peak, and PWV values
- 9-month follow-up data will include re assessments of VO_2_ peak and PWV values 9 months after cessation of formal exercise interventions.

Exercise intervention

Exercise interenvention in both the AT and RT groups will have been completed for 12weeks as per the ExeRT trial (18). Participants in this follow-up study will attend their 9-month follow-up assessment (AT and RT groups), or no intervention (UC group).

Participants randomized into either the AT or RT groups will have participated in 12 weeks of supervised, outpatient exercise sessions, phone follow up and supplemental home exercise diaries. Aerobic training participants had individualised programmes created with 80% Heart rate reserve targets and use of polar heart rate watches to monitor performance.

Participants in the RT group will have participated in 12 weeks of supervised outpatient exercise sessions with phone follow up and supplemental Home exercise diaries. Exercise programmes where individualized using 80% 1 repetition max.

Usual care (UC) is defined as the standard exercise/ physical activity counselling renal transplant recipients receive from their nephrologist or renal nurse during routine outpatient visits. The two sites selected in this study both received similar standard education from the post-transplant team.

**7. Data analysis:**

- Statistical consultation was gained from Trevor Murrells (Statistician Kings College London University) and supervisor Dr Sharlene Greenwood.
- Descriptive analysis of data will be completed- including dropouts.
- Data will be assessed for Normality
- Between group differences will be assessed via ANCOVA analysis allowing for variables influencing arterial stiffness such as age, length of time on dialysis pre transplant and baseline values as co-variates

Within group differences will be assessed via t-tests or non-parametric equivalents depending on the distribution of the data.

**8. References:**

1. Greenwood SA, Lindup H, Taylor K, Koufaki P, Rush R, Macdougall IC, et al. Evaluation of a pragmatic exercise rehabilitation programme in chronic kidney disease. Nephrology Dialysis Transplantation. 2012;27(suppl 3):iii126-iii34.

2. Johansen KL, Painter P. Exercise in individuals with CKD. American Journal of Kidney Diseases. 2012;59(1):126-34.

3. Heiwe S, Jacobson SH. Exercise training for adults with chronic kidney disease. The Cochrane database of systematic reviews. 2011;10(10).

4. Koufaki P, Greenwood SA, Macdougall IC, Mercer TH. Exercise therapy in individuals with chronic kidney disease: a systematic review and synthesis of the research evidence. Annu Rev Nurs Res. 2013;31:235-75.

5. Nielens H, Lejeune TM, Lalaoui A, Squifflet JP, Pirson Y, Goffin E. Increase of physical activity level after successful renal transplantation: a 5 year follow‐up study. Nephrology Dialysis Transplantation. 2001;16(1):134-40.

6. Painter P, Krasnoff JB, Kuskowski M, Frassetto L, Johansen KL. Effects of modality change and transplant on peak oxygen uptake in patients with kidney failure. American Journal of Kidney Diseases. 2011;57(1):113-22.

7. Macdonald JH, Kirkman D, Jibani M. Kidney transplantation: a systematic review of interventional and observational studies of physical activity on intermediate outcomes. Adv Chronic Kidney Dis. 2009;16(6):482-500.

8. Sietsema KE, Amato A, Adler SG, Brass EP. Exercise capacity as a predictor of survival among ambulatory patients with end-stage renal disease. Kidney international. 2004;65(2):719-24.

9. Van Den Ham EC, Kooman JP, Schols AM, Nieman FH, Does JD, Franssen FM, et al. Similarities in skeletal muscle strength and exercise capacity between renal transplant and hemodialysis patients. American journal of transplantation. 2005;5(8):1957-65.

10. Padilla J, Krasnoff J, DaSilva M, Hsu C-Y, Frassetto L, Johansen KL, et al. Physical functioning in patients with chronic kidney disease. Journal of nephrology. 2008;21(4):550-9.

11. Chan W, Bosch JA, Jones D, Kaur O, Inston N, Moore S, et al. Predictors and consequences of fatigue in prevalent kidney transplant recipients. Transplantation. 2013;96(11):987-94.

12. Goedendorp MM, Hoitsma AJ, Bloot L, Bleijenberg G, Knoop H. Severe fatigue after kidney transplantation: a highly prevalent, disabling and multifactorial symptom. Transplant International. 2013;26(10):1007-15.

13. Painter P, Hanson P, Messer-Rehak D, Zimmerman S, Glass N. Exercise tolerance changes following renal transplantation. American Journal of Kidney Diseases. 1987;10(6):452-6.

14. Habedank D, Kung T, Karhausen T, Von Haehling S, Doehner W, Schefold JC, et al. Exercise capacity and body composition in living-donor renal transplant recipients over time. Nephrology Dialysis Transplantation. 2009;24(12):3854-60.

15. Painter PL, Hector L, Ray K, Lynes L, Dibble S, Paul SM, et al. A randomized trial of exercise training after renal transplantation. Transplantation. 2002;74(1):42-8.

16. van den Ham EC, Kooman JP, Schols AM, Nieman FH, Does JD, Akkermans MA, et al. The functional, metabolic, and anabolic responses to exercise training in renal transplant and hemodialysis patients. Transplantation. 2007;83(8):1059-68.

17. Kouidi E, Vergoulas G, Anifanti M, Deligiannis A. A randomized controlled trial of exercise training on cardiovascular and autonomic function among renal transplant recipients. Nephrology Dialysis Transplantation. 2013;28(5):1294-305.

18. Greenwood SA, Koufaki P, Mercer TH, Rush R, O’Connor E, Tuffnell R, Lindup H, Haggis L, Dew T, Abdulnassir L, Nugent E. Aerobic or resistance training and pulse wave velocity in kidney transplant recipients: a 12-week pilot randomized controlled trial (the Exercise in Renal Transplant [ExeRT] Trial). American Journal of Kidney Diseases. 2015 Oct 31;66(4):689-98.
